# Opposing Cholinergic and Serotonergic Modulation of Layer 6 in Prefrontal Cortex

**DOI:** 10.3389/fncir.2017.00107

**Published:** 2018-01-04

**Authors:** Daniel W. Sparks, Michael K. Tian, Derya Sargin, Sridevi Venkatesan, Katheron Intson, Evelyn K. Lambe

**Affiliations:** ^1^Department of Physiology, University of Toronto, Toronto, ON, Canada; ^2^Department of Pharmacology and Toxicology, University of Toronto, Toronto, ON, Canada; ^3^Department of Obstetrics and Gynecology, University of Toronto, Toronto, ON, Canada; ^4^Department of Psychiatry, University of Toronto, Toronto, ON, Canada

**Keywords:** acetylcholine, serotonin, attention, stress, prefrontal, corticothalamic, optogenetics, electrophysiology

## Abstract

Prefrontal cortex is a hub for attention processing and receives abundant innervation from cholinergic and serotonergic afferents. A growing body of evidence suggests that acetylcholine (ACh) and serotonin (5-HT) have opposing influences on tasks requiring attention, but the underlying neurophysiology of their opposition is unclear. One candidate target population is medial prefrontal layer 6 pyramidal neurons, which provide feedback modulation of the thalamus, as well as feed-forward excitation of cortical interneurons. Here, we assess the response of these neurons to ACh and 5-HT using whole cell recordings in acute brain slices from mouse cortex. With application of exogenous agonists, we show that individual layer 6 pyramidal neurons are bidirectionally-modulated, with ACh and 5-HT exerting opposite effects on excitability across a number of concentrations. Next, we tested the responses of layer 6 pyramidal neurons to optogenetic release of endogenous ACh or 5-HT. These experiments were performed in brain slices from transgenic mice expressing channelrhodopsin in either ChAT-expressing cholinergic neurons or Pet1-expressing serotonergic neurons. Light-evoked endogenous neuromodulation recapitulated the effects of exogenous neurotransmitters, showing opposing modulation of layer 6 pyramidal neurons by ACh and 5-HT. Lastly, the addition of 5-HT to either endogenous or exogenous ACh significantly suppressed the excitation of pyramidal neurons in prefrontal layer 6. Taken together, this work suggests that the major corticothalamic layer of prefrontal cortex is a substrate for opposing modulatory influences on neuronal activity that could have implications for regulation of attention.

## Introduction

The medial prefrontal cortex is essential for higher cognitive functions such as attention (Miller and Cohen, 2001; Knudsen, 2007; Logue and Gould, 2014). Although debate exists concerning the relationship of frontal brain areas between species in respect to structure and function, there is general agreement that the medial frontal portion of the brain is essential for executive functions, such as attention (Uylings et al., 2003; Bicks et al., 2015; Carlen, 2017). This is supported by the fact that lesions to this region impair performance on attention tasks across species, demonstrated by work in humans (Szczepanski and Knight, 2014), nonhuman primates (Rossi et al., 2009) and rodents (Muir et al., 1996; Granon et al., 1998). The deepest layer of prefrontal cortex, layer 6, is emerging as a key player in attentional control (Zikopoulos and Barbas, 2006; Béhuret et al., 2015; Wimmer et al., 2015). This layer sends extensive projections to thalamic nuclei (Mercer et al., 2005; Watts and Thomson, 2005; West et al., 2006; Zikopoulos and Barbas, 2006; Thomson, 2010) and to local circuit interneurons in cerebral cortex (Olsen et al., 2012; Tian et al., 2016). The corticothalamic connections are critical for top-down control of attentional processes (Miller and Cohen, 2001; Alitto and Usrey, 2003; Sherman, 2007; Wallace and Bertrand, 2013) and the corticocortical connections mediate cortical gain control (Olsen et al., 2012; Kim et al., 2016). The combination of these functions place layer 6 of prefrontal cortex as a hub connecting two important networks in control of attention. The importance of understanding the complexities of layer 6 modulation is emphasized by recent work showing that the frequency of corticothalamic neuronal firing determines whether it will inhibit or excite the thalamus (Crandall et al., 2015).

Deep layers of medial prefrontal cortex receive input from a number of neurotransmitter systems that modulate attentional processes (Logue and Gould, 2014; Meunier et al., 2017), with two prominent ones being acetylcholine (Ach; Robbins and Roberts, 2007; Bentley et al., 2011; Hasselmo and Sarter, 2011) and serotonin (5-HT; Amargós-Bosch et al., 2004; de Almeida and Mengod, 2008; Mengod et al., 2015; Muzerelle et al., 2016). Cholinergic projections to the mPFC arise from the nucleus basalis of the basal forebrain (Dunnett et al., 1991; Muir et al., 1993; Pang et al., 1993; McGaughy et al., 2002; Logue and Gould, 2014) and are associated with enhanced performance on attentional tasks (Himmelheber et al., 2000; Passetti et al., 2000; Sarter et al., 2001; Wallace and Bertrand, 2013). Disruptions to the cholinergic system interfere with attention (Robbins et al., 1989; Muir et al., 1992, 1993; Pang et al., 1993; Voytko et al., 1994; McGaughy et al., 2002; Dalley et al., 2004; Newman and McGaughy, 2008; Hasselmo and Sarter, 2011). Prefrontal cortex also receives 5-HT projections from the midbrain dorsal raphe nucleus (Amargós-Bosch et al., 2004; Celada et al., 2013; Logue and Gould, 2014; Leiser et al., 2015; Mengod et al., 2015; Muzerelle et al., 2016). Brain serotonergic activity has the opposite effect on attention than ACh. Increased 5-HT levels negatively affect performance on attention tasks (Ramaekers et al., 1995; Riedel et al., 1999, 2005; Schmitt et al., 2002; Wingen et al., 2008; Graf et al., 2013; Golub et al., 2017), while reductions in 5-HT enhance attention (Schmitt et al., 2000; Gallagher et al., 2003; Wingen et al., 2007). While it has been suggested that ACh and 5-HT can alter performance in the same attentional task (Jäkälä et al., 1992), it is not yet clear that there is a direct cortical interaction between these two modulatory systems in attentional processes (Steckler and Sahgal, 1995).

If ACh and 5-HT engage in a “tug-of-war” over attention, layer 6 of prefrontal cortex is an excellent candidate location for this interaction. Layer 6 pyramidal neurons are excited by ACh (Kassam et al., 2008; Bailey et al., 2010; Guillem et al., 2011; Tian et al., 2011; Poorthuis et al., 2013) and inhibited by 5-HT (Tian et al., 2016), with effects persisting in the presence of synaptic blockers, suggesting that ACh and 5-HT act directly on postsynaptic receptors on the same neurons. In prefrontal layer 6, there is abundant expression of the α4 and β2 subunits of high affinity nicotinic ACh receptors, as well as the α5 accessory subunit (Wada et al., 1989). Similarly, there is prominent expression of 5-HT_1A_ receptors (Amargós-Bosch et al., 2004; Santana et al., 2004). However, it is not known whether the same neurons are bidirectionally modulated, nor how ACh and 5-HT in combination would affect the physiology of individual layer 6 pyramidal neurons. These questions are important because the cholinergic and serotonergic modulatory systems are both active during waking (Buzsaki et al., 1988; Jacobs and Azmitia, 1992) and are dynamically regulated in response to environmental stimuli (5-HT: Ranade and Mainen, 2009; Cohen et al., 2015; ACh: Buzsaki et al., 1988; Détári and Vanderwolf, 1987; Parikh et al., 2007; Sarter et al., 2016). Prefrontal 5-HT is increased by acute stressors (Fujino et al., 2002; Bland et al., 2003), which are known to impair attentional performance (Sänger et al., 2014). Yet rodent cognitive testing may underestimate the role of prefrontal 5-HT on attention, as typical training and testing paradigms (Winstanley et al., 2003; Bari et al., 2008) employ chronic stress conditions that would reduce levels of prefrontal 5-HT, including food restriction followed by rewards (Chandler-Laney et al., 2007; Fallon et al., 2007) and prolonged periods of single housing (Sargin et al., 2016), leading to an underestimation of potential effects of 5-HT on attention during acute stressors. Of note, human work suggests that social and emotional context will increase 5-HT effects on cognition (Osinsky et al., 2008; Daly et al., 2010; Beacher et al., 2011; Elliott et al., 2011; Frodl et al., 2015).

Here, we examined how the cholinergic and serotonergic systems interact in prefrontal cortex at the level of layer 6 neuronal physiology. Initial experiments examined whether individual layer 6 neurons are modulated by both ACh and 5-HT. Next, we investigated the effects of endogenous neurotransmitter release from cholinergic or serotonergic terminals. Finally, we studied the interaction between these modulators by assessing changes in neuronal responses to ACh during exposure to 5-HT. Taken together, this work begins to assess the outcome of combined cholinergic and serotonergic regulation of a prefrontal layer at the heart of attention. This could have significant implications for understanding how attentional processes in prefrontal cortex are modulated during times of heightened stress or emotion.

## Materials and Methods

### Experimental Animals/Brain Slice Preparation

Guidelines of the Canadian Council on Animal Care were followed, and all experimental procedures were approved by the Faculty of Medicine Animal Care Committee at the University of Toronto. Wild-type mice on a C57BL/6 background were used for experiments assessing the effects of exogenous ACh and 5-HT on neuronal activity. To study the effects of endogenous ACh release on neural activity, mice that heterozygously express blue-light sensitive channelrhodopsin in ChAT-containing cholinergic projections neurons (ChAT-ChR2-YFP BAC) on a C57BL/6 background were used (Zhao et al., 2011; Ivanova et al., 2016), permitting blue light stimulation to trigger the release of ACh from presynaptic terminals. Additional experiments were performed in mice heterozygous for ChATcre (ChAT-IRES-Cre, Rossi et al., 2011; Chen et al., 2016) and for Ai32 (RCL-ChR2(H134R)/EYFP, Madisen et al., 2012; O’Neill et al., 2017), a second mouse line that led to expression of blue-light sensitive channelrhodopsin in ChAT-containing cholinergic projections neurons. To assess the effects of endogenous 5-HT release on neural activity, mice heterozygous for Pet1cre (Tg(Fev-cre)1Esd, Scott et al., 2005) and homozygous for Ai32 (RCL-ChR2(H134R)/EYFP) on a C57BL/6 background were used, permitting blue light stimulation to release 5-HT from presynaptic terminals. A small subset of Pet1cre mice heterozygous for Ai32 were also tested. As layer 6 neurons in these mice did not detect an effect of light stimulation, we followed up with recording from 5-HT neurons in dorsal raphe in these mice. At 3 weeks of age mice were weaned, separated based on sex, and group housed (2–4 mice per cage) and given *ad libitum* access to food and water on a 12-h light/dark cycle with lights on at 7 AM.

### Electrophysiology

Electrophysiology experiments were performed in acute brain slices obtained from adult male mice (mean ± SE; postnatal day 110 ± 5, *n* = 36 mice). After deep anesthesia with chloral hydrate (400 mg/kg), mice were decapitated and their brains were quickly extracted and chilled in 4°C sucrose ACSF (254 mM sucrose, 10 mM D-glucose, 24 mM NaHCO_3_, 2 mM CaCl_2_, 2 mM MgSO_4_, 3 mM KCl, 1.25 mM NaH_2_PO_4_; pH 7.4). A Dosaka linear slicer (SciMedia, Costa Mesa, CA, USA) was used to obtain 400 μM thick coronal brain slices of prefrontal cortex (range 2.34–0.74 from Bregma; Paxinos and Franklin, 2001), and for a small subset of mice coronal slices of dorsal raphe (range −4.48 to −4.84 from Bregma; Paxinos and Franklin, 2001), which recovered for ~2 h in regular ACSF (128 mM NaCl, 10 mM D-glucose, 26 mM NaHCO_3_, 2 mM CaCl_2_, 2 mM MgSO_4_4, 3 mM KCl, 1.25 mM NaH_2_PO_4_; pH 7.4). To maintain synthesis of 5-HT (Liu et al., 2005), brain slices from Pet1cre/Ai32^+/−^ and Pet1cre/Ai32^+/+^ were recovered and recorded in the presence of L-tryptophan (2.5 μM for dorsal raphe slices; 30 μM for prefrontal slices).

For whole cell patch clamp recording, brain slices were placed in a perfusion chamber on the stage of a BX50W1 microscope (Olympus, Tokyo, Japan), perfused with oxygenated ACSF (95%O_2_, 5%CO_2_) at a rate of 3–4 ml/minute at 30°C for cortical slices and room temperature for dorsal raphe slices. Layer 6 pyramidal neurons in the prelimbic and cingulate regions of the medial prefrontal cortex were identified based on morphological characteristics (pyramidal shape, large cell body, orientation of apical dendrite; see Contreras, 2004; Andjelic et al., 2009; van Aerde and Feldmeyer, 2015). Recording electrodes (2–4 MΩ) filled with 5 mM KCl, 2 mM MgCl_2_, 4 mM K_2_-ATP, 0.4 mM Na_2_-GTP, 10 mM Na_2_-phosphocreatine and 10 mM HEPES buffer and with pH adjusted to 7.3 using KOH were used to patch layer 6 pyramidal neurons. Recordings were obtained using an EPC10 (HEKA Electronik, Lambrecht/Pfalz, Germany). All data were acquired at 20 kHz and low pass filtered at 3 kHz using pClamp software (Molecular Devices, Palo Alto, CA, USA) and corrected for the liquid junction potential (14 mV). For the small subset of recordings in the dorsal raphe, the electrophysiological properties of the GFP-positive 5-HT neurons we observed were: resting membrane potential (RMP; −64 ± 2 mV), input resistance (750 ± 81 MΩ), and action potential amplitude (94 ± 5 mV). The electrophysiological properties of prefrontal layer 6 pyramidal neurons from the different mouse lines are illustrated in Table 1.

**Table 1:** Electrophysiological properties of prefrontal layer 6 pyramidal neurons in each mouse line.

Mouse line	*n*	RMP (mV)	R_in_ (MΩ)	Spike ampl (mV)	Threshold (mV)
WT	32	−89 ± 1	166 ± 16	81 ± 2	−53 ± 1
ChAT-ChR2	68	−81 ± 4	186 ± 10	76 ± 2	−51 ± 1
ChATCre/Ai32^+/−^	8	−86 ± 1	126 ± 8	81 ± 5	−45 ± 2
Pet1Cre/Ai32^+/+^	35	−87 ± 1	137 ± 12	81 ± 2	−50 ± 1

*Intrinsic neuronal properties illustrated are resting membrane potential (RMP), input resistance (R_m_), spike amplitude (ampl) and spike threshold. Data shown as mean ± SE*.

We employed several different electrophysiological strategies to examine the co-modulation of layer 6 pyramidal neurons by ACh and 5-HT. Initial experiments probed the sensitivity of individual neurons to ACh and 5-HT using relatively strong exogenous stimulation (1 mM ACh for 15 s and 10 μM 5-HT for 30 s). Resulting currents were measured in voltage-clamp at a holding potential of −75 mV. Next, we tested the sensitivity of layer 6 neurons to modulation that is potentially more physiologically-relevant, applying lower concentrations of exogenous ACh and 5-HT (10 μM ACh or 1 μM 5-HT, 30 s). For these experiments, we aimed to simulate how activation of cholinergic or serotonergic inputs to the prefrontal cortex might affect the firing rate of layer 6 neurons. To this end, we recorded from layer 6 pyramidal neurons in current clamp and delivered trains of brief depolarizing stimuli (5 ms pulses at 5 Hz for 4 s; repeated every 10 s). Stimulation intensity was selected to be “peri-threshold,” yielding ~50% success at eliciting action potentials in a replicable pattern across baseline trials. Changes in firing frequency were then recorded following application of either ACh or 5-HT under these conditions.

To test the effects of *endogenously*-released ACh or 5-HT, we used optogenetic experiments in which channelrhodopsin-expressing terminals of either ChAT or Pet1-expressing neurons were stimulated with blue (473 nm; 2–6 mW) LED light using an optic fiber (Thorlabs, Newton, NJ, USA) on a mechanical manipulator (Narishige International, East Meadow, NY, USA) targeted onto layer 6 pyramidal neurons or a microscope-mounted collimated LED (Thorlabs). In brain slices of ChAT/ChR2 and ChATcre/Ai32^+/−^ mice, neurons were held at subthreshold membrane potentials (−70 to −65 mV) and light (5 ms pulses at 10 Hz for 3 s) was used to induce ACh release. In Pet1Cre/Ai32^+/+^ mice, neurons were depolarized to supra-threshold levels to elicit action potential firing (2–3 Hz) and light (5 ms pulses, 10 Hz, 5 s) used to assess the suppression of action potential firing by endogenous 5-HT release. Additional experiments applied peri-threshold trains of depolarizing stimuli to layer 6 neurons in either ChAT/ChR2 or Pet1Cre/Ai32 mice to assess the effects of endogenous neurotransmitter release on neuronal firing rate. Finally, the inhibitory effects of low levels of 5-HT were tested against the depolarization elicited by the strongest level of light-evoked ACh release (5 ms pulses, 10 Hz), as well as the interaction between higher levels of exogenous 5-HT and ACh.

### Pharmacology

For the exogenous cholinergic and serotonergic stimulation, neuronal current responses were assessed by bath application of ACh (acetylcholine chloride, Sigma-Aldrich, Oakville, ON, Canada) and 5-HT (serotonin creatinine sulfate, Sigma-Aldrich, St. Louis, MO) in ACSF. Neurons were tested with strong stimulation (1 mM ACh for 15 s; 10 μM 5-HT for 30 s) or milder stimulation (10 μM ACh for 30 s; 1 μM 5-HT for 30 s) to approximate endogenous conditions more closely. For the optogenetic experiments, the role of different cholinergic receptor subtypes in mediating the light-evoked ACh response was assessed using the α4β2 nicotinic ACh receptor antagonist DHβE (Sigma-Aldrich, 3 μM), and the muscarinic receptor antagonist atropine (Sigma-Aldrich, 200 nM). The role of different 5-HT receptor subtypes in the light-evoked 5-HT response was assessed using the 5-HT_1A_ receptor antagonist WAY100635 (Tocris, Bristol, UK; 30 nM).

### Statistical Analysis

All data are expressed as mean ± SE. Recordings were analyzed using Clampfit software (Molecular Devices) and statistically analyzed using GraphPad Prism 6 software (GraphPad Software, La Jolla, CA, USA) using paired or unpaired Student’s *t*-tests with a significance level of *p* < 0.05.

## Results

### Opposite Cholinergic and Serotonergic Modulation of Prefrontal Layer 6 Neurons

**Figure 1:**
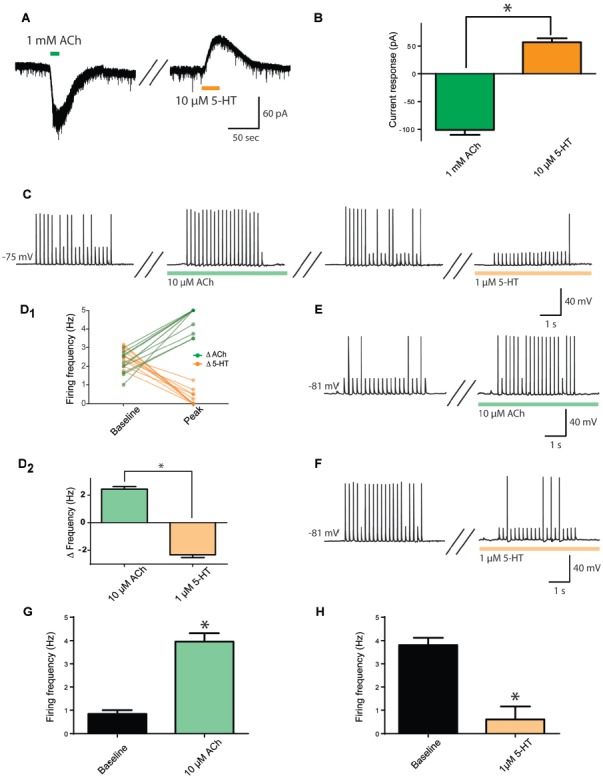
Opposing cholinergic and serotonergic modulation of prefrontal layer 6 neurons. **(A)** Example voltage-clamp recording showing inward current elicited by strong acetylcholine (ACh) stimulation (1 mM) and outward current elicited by strong serotonin (5-HT; 10 μM) stimulation in the same layer 6 neuron. **(B)** Bar graph summarizing inward and outward currents elicited by ACh and 5-HT respectively in layer 6 pyramidal neurons (*t*_9_ = 13.7, *p* < 0.0001). **(C)** To assess the effects of lower stimulation by ACh and 5-HT on firing frequency, a train of peri-threshold current pulses was delivered to layer 6 pyramidal neurons. Example neuron shows the frequency of action potentials elicited compared across baseline, ACh (10 μM), washout, and 5-HT (1 μM) conditions. **(D)** Linked-data plot **(D_1_)** and bar graph **(D_2_)** summarize the change in frequency of action potentials fired in response to ACh and 5-HT respectively in layer 6 pyramidal neurons (*t*_11_ = 15.3, *p* < 0.0001). The strength of this modulation raises the possibility of ceiling and floor effects. **(E)** Therefore, to probe further the ability of ACh (10 μM) to enhance action potential firing, a train of current pulses with lower spike probability was used, as shown in this example. **(F)** Furthermore, to probe further the ability of 5-HT (1 μM) to suppress action potential firing, a train of current pulses with greater spike probability was used, as shown in this example. **(G)** Bar graph summarizing the spike frequency enhancing effects of a low level of exogenous ACh (*t*_18_ = 7.57, *p* < 0.0001). **(H)** Bar graph showing the spike frequency suppressing effects of a low level of 5-HT (*t*_6_ = 5.4, *p* = 0.0016). *Denotes *p* < 0.05. Darker green and orange indicate higher concentration of ACh and 5-HT (1 mM and 10 μM, respectively), while lighter green and orange indicate lower concentration of ACh and 5-HT (10 μM and 1 μM, respectively).

First, we investigated whether the same prefrontal layer 6 pyramidal neurons respond both to ACh and to 5-HT, using bath application of these neurotransmitters and measuring current responses in voltage clamp. As illustrated in Figures 1A,B, ACh (1 mM) elicited large inward currents (−101 ± 9 pA) and, after a 5 min washout period, 5-HT (10 μM) elicited moderate outward currents (57 ± 7 pA). These responses were significantly different (*t*_9_ = 13.7, *p* < 0.0001), but there was no significant correlation between the size of ACh and 5-HT responses in the same neurons (*r* = 0.18, *p* = 0.6, *n* = 10). A lower concentration of ACh (10 μM), elicited smaller inward currents (−7.6 ± 2 pA, *n* = 13), while a lower concentration of 5-HT (1 μM), elicited smaller outward currents (18.0 ± 4 pA, *n* = 8). The statistically significant difference between responses to ACh and 5-HT was maintained (*t*_19_ = 6.9, *p* < 0.0001).

With the ultimate goal of assessing how endogenous ACh and 5-HT modulate layer 6 neuronal activity, we developed an experimental paradigm more sensitive to lower neurotransmitter levels. To accomplish this, we determined the peri-threshold current amplitude experimentally and administered trains of current injection in current clamp from rest (5 ms pulses at 5 Hz for 4 s, average baseline firing frequency: 2.4 ± 0.1 Hz). This level would permit bidirectional modification of action potential firing. As illustrated in Figures 1C,D_1,2_, ACh and 5-HT had opposing and significantly different effects on action potential firing in response to the current train in the same neurons (*t*_11_ = 15.3, *p* < 0.0001, *n* = 12), with ACh (10 μM) significantly increasing action potential firing rate by 2.4 ± 0.2 Hz and 5-HT (1 μM) significantly decreasing action potential firing rate by 2.3 ± 0.2 Hz. The strength of this modulation raised the possibility of ceiling and floor effects. Therefore, as illustrated in Figures 1E–H, we tested cholinergic excitation using a lower initial spike success level, finding a significant increase in action potential firing frequency (baseline, 0.8 ± 0.2; ACh, 4.0 ± 0.4 Hz; *t*_18_ = 7.57, *p* < 0.0001, *n* = 19) and serotonergic inhibition using a higher initial spike success level, finding a significant decrease in action potential firing frequency (baseline, 3.8 ± 0.3; 5-HT, 0.6 ± 0.6 Hz; *t*_6_ = 5.4, *p* = 0.002, *n* = 7). These results illustrate that ACh and 5-HT have opposing effects on the excitability of layer 6 prefrontal neurons.

### Endogenous Modulation of Action Potential Firing in Prefrontal Layer 6 Neurons

**Figure 2:**
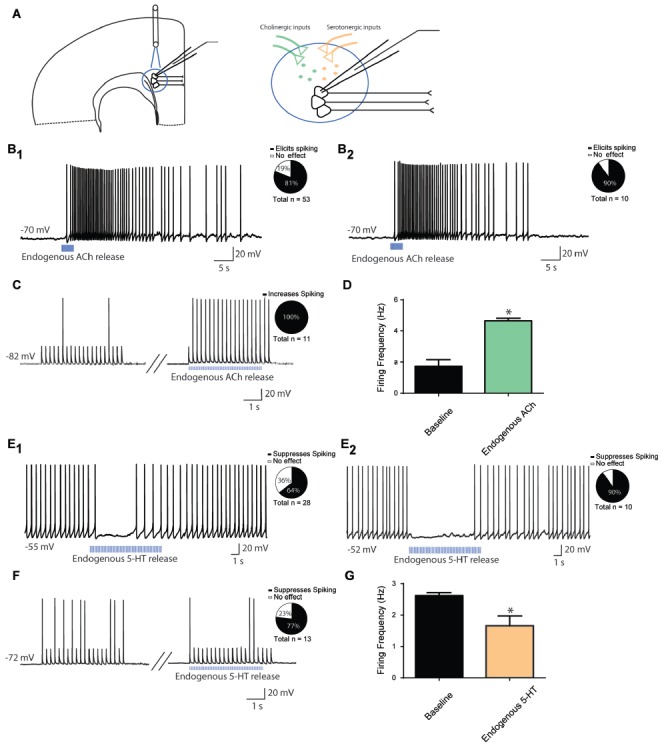
Endogenous modulation of action potential firing in prefrontal layer 6 neurons. **(A)** Schematic diagram illustrating light-evoked ACh or 5-HT release onto layer 6 pyramidal neurons from presynaptic terminals. **(B)** A train of optogenetic stimuli (5 ms pulses, 10 Hz) to release ACh can elicit depolarization and action potential spiking in layer 6 neurons in cortical slices of mice with channelrhodopsin in axonal fibers from ChAT-expressing ACh neurons. For this experiment, neurons were injected with constant electrical current to bring them to a membrane potential of −70 mV **(B_1_)**. Pie chart illustrates that action potentials were elicited in 43/53 neurons. ACh-elicited action potential firing persisted in the presence of synaptic blockers CNQX, APV and picrotoxin **(B_2_)**. Pie chart illustrates that action potentials were evoked in in 9/10 neurons. **(C)** In combination with a train of peri-threshold electrical pulses, optogenetic release of ACh increases the probability of action potential firing. Pie chart illustrates that action potential frequency was increased in 11/11 neurons. **(D)** Bar graph showing that spike frequency was significantly increased by optogenetic release of endogenous ACh (*t*_10_ = 6.9, *p* < 0.0001). **(E)** A train of optogenetic stimuli (5 ms pulses, 10 Hz) to release 5-HT can reduce action potential firing in layer 6 neurons in cortical slices of mice with channelrhodopsin in axonal fibers from Pet1cre-expressing 5-HT neurons **(E_1_)**. For this experiment, neurons were injected with constant electrical current to bring them to spiking. Pie chart illustrates that action potentials were suppressed in 18/28 neurons. The 5-HT elicited suppression persisted in the presence of synaptic blockers CNQX, APV and picrotoxin **(E_2_)**. Pie chart illustrates that action potentials were suppressed in 9/10 neurons. **(F)** In combination with a train of peri-threshold electrical pulses, optogenetic stimuli to release 5-HT decreases the probability of action potential firing. Pie chart illustrates that action potential frequency was decreased in 10/13 neurons. **(G)** Bar graph showing a small but significant decrease in spike frequency upon optogenetic release of 5-HT (*t*_12_ = 3.3, *p* = 0.007). *Denotes *p* < 0.05.

To test the effects of endogenous ACh or 5-HT release on the firing activity of layer 6 pyramidal neurons, we employed transgenic mouse models that allowed us to use light to stimulate ACh or 5-HT release onto layer 6 pyramidal neurons (Figure 2A). Note, levels of channelrhodopsin expressed in afferents correspond to the different cholinergic and serotonergic mouse lines used. As such, response to light stimulation shows that modulation is possible, but does not set an upper bound on its strength, and lack of response does not rule out a modulatory role for those afferents.

As illustrated in Figure 2B_1_, light stimulation of cholinergic terminals in brain slices of ChAT-ChR2 mice led to action potential firing in 43/53 (*n* = 53) layer 6 pyramidal neurons held at subthreshold membrane potentials. Of note, optogenetic stimulation of cholinergic afferents in a different transgenic line (ChATcre/Ai32^+/−^) had similar effects, with light stimulation inducing action potential firing in 6/8 neurons (*n* = 8, not shown). This effect persisted in the presence of synaptic blockers CNQX, APV and picrotoxin in 9/10 neurons (*n* = 10, Figure 2B_2_). Pharmacological manipulation of nicotinic and muscarinic ACh receptors, respectively with DHβE (3 μM) and/or atropine (200 nM), either individually or in tandem, both contributed to endogenous ACh-induced spiking. DHβE blocked spiking in 5/7 neurons, while atropine blocked spiking in 5/7 neurons. Co-application of DHβE and atropine blocked spiking in 5/5 neurons tested. These findings are consistent with a role for both subtypes of ACh receptors in mediating the response of layer 6 pyramidal neurons to exogenous ACh (Kassam et al., 2008; Bailey et al., 2010; Tian et al., 2011). Next, we examined how light-evoked endogenous release of ACh affects firing activity during trains of depolarizing current injection, in order to assess the consequences of endogenous ACh for prefrontal neurons to follow stimuli. Optogenetic stimulation of cholinergic afferents led to a significant increase in neuronal firing rate following trains of current injection in neurons that were sitting at RMP (baseline, 1.7 ± 0.4; endogenous ACh, 4.7 ± 0.2 Hz; *t*_10_ = 6.9, *p* < 0.0001), with an increase in firing rate occurring in 11/11 neurons tested (*n* = 11, Figures 2C,D).

Although 5-HT terminals are found in abundance in the prefrontal cortex, early experiments in Pet1cre/Ai32^+/−^ mice did not detect a response to light in the cortex (*n* = 6), despite robust light-elicited inward currents in dorsal raphe 5-HT cells (1.4 ± 0.4 nA, *n* = 11; response to a single 5 ms flash of light). Therefore, we bred this line to be homozygous for Ai32 (Pet1cre/Ai32^+/+^) with the goal of increasing the channelrhodopsin expression in serotonergic afferents. In these Pet1cre/Ai32^+/+^ mice, optogenetic stimulation of serotonergic afferents in prefrontal slices had detectable electrophysiological effects. We injected depolarizing current to elicit stable baseline action potential firing (2–3 Hz) in order to measure the degree of inhibition mediated by endogenous 5-HT release. Using this paradigm, light stimulation of serotonergic afferents inhibited action potential firing in 18/28 neurons (*n* = 28, Figure 2E_1_). This effect persisted in the presence of synaptic blockers CNQX, APV and picrotoxin in 9/10 neurons (*n* = 10, Figure 2E_2_). Consistent with exogenous serotonergic modulation (Tian et al., 2016), the light evoked responses in brain slices from Pet1cre/Ai32^+/+^ mice were sensitive to suppression by an antagonist of 5-HT receptors. Pharmacological manipulation of 5-HT_1A_ receptors with WAY100635 (30 nM) completely suppressed the light-induced inhibition in 5/6 neurons tested. Finally, we examined how endogenous release of 5-HT affects firing activity in response to current injection at RMP, simulating how 5-HT may modulate prefrontal layer 6 neuronal activity in response to inputs. Optogenetic stimulation of serotonergic afferents led to a significant decrease in neuronal firing rate (baseline, 2.6 ± 0.1 Hz; endogenous 5-HT, 1.7 ± 0.3 Hz; *t*_12_ = 3.3, *p* = 0.007), with a decrease in firing rate occurring in 10/13 neurons tested (*n* = 13, Figures 2F,G). These results suggest that endogenous ACh and 5-HT modulate layer 6 prefrontal neuron excitability in similar directions to exogenous bath application, with endogenous ACh increasing and endogenous 5-HT decreasing neuronal excitability.

### Interaction of Cholinergic and Serotonergic Influences on Layer 6 Neuronal Activity

**Figure 3:**
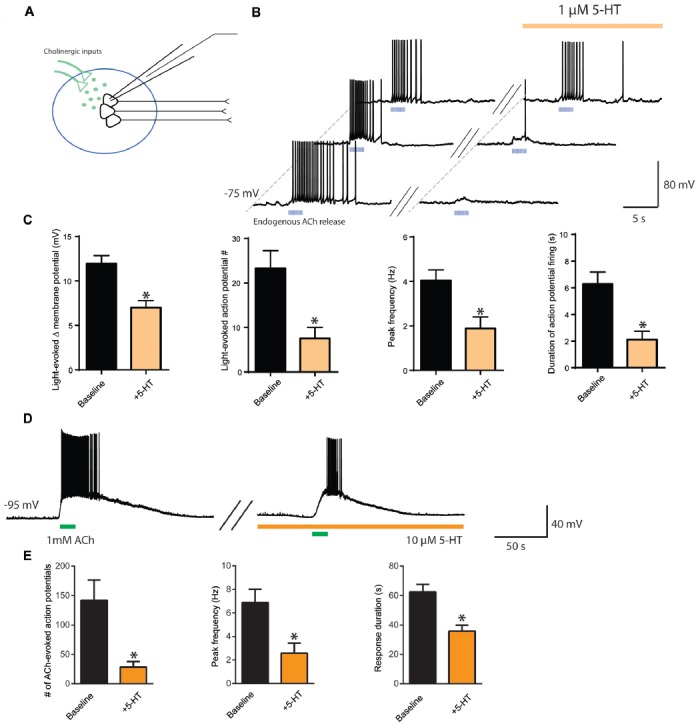
Interaction of cholinergic and serotonergic influences on layer 6 neuronal activity. 5-HT inhibits ACh-induced facilitation of action potential firing. **(A)** Schematic illustrating light-evoked stimulation of cholinergic fibers synapsing onto layer 6 pyramidal neurons in medial prefrontal cortex. **(B)** Three different example layer 6 pyramidal neurons demonstrating that the action potential firing evoked by endogenous ACh (5 ms pulses, 10 Hz) is inhibited by application of a low level of exogenous 5-HT (1 μM). **(C)** Bar charts show the significant reductions exerted by 5-HT on the following parameters of the layer 6 excitation elicited by optogenetic release of endogenous ACh: change in membrane potential (*t*_24_ = 4.7, *p* < 0.0001), number of action potentials (*t*_23_ = 5.2, *p* < 0.0001), peak spike frequency (*t*_22_ = 6.0, *p* < 0.0001), and duration of action potential firing (*t*_24_ = 6.3, *p* < 0.0001). **(D)** Example neuron showing that the stronger excitation of layer 6 pyramidal neurons through application of exogenous ACh remains susceptible to attenuation by 5-HT. **(E)** Bar charts show the significant reductions exerted by 5-HT on the following parameters of layer 6 excitation is inhibited by 5-HT: number of action potentials (*t*_11_ = 4.0, *p* = 0.0021), peak frequency (*t*_11_ = 7.4, *p* < 0.0001) and response duration (*t*_12_ = 5.27, *p* = 0.0003). *Denotes *p* < 0.05. Darker orange indicates a higher concentration of 5-HT (10 μM), while lighter orange indicates a lower concentration of 5-HT (1 μM).

To observe more directly the interaction between ACh and 5-HT and their opposing influences on neuronal activity in layer 6 pyramidal neurons, we next optogenetically stimulated cholinergic afferents (Figure 3A) before and during bath application of 5-HT (1 μM) to simulate the coincident activity of ACh and 5-HT on layer 6 neurons. The presence of 5-HT inhibited the excitability normally induced by ACh light stimulation (*n* = 25, Figures 3B,C). There was a significant reduction in ACh-elicited depolarization (baseline, 12.0 ± 0.9 mV; 5-HT, 7.0 ± 0.8 mV; *t*_24_ = 4.7, *p* < 0.0001), a significant decrease in the number of action potentials evoked (baseline, 23 ± 4 spikes; 5-HT, 8 ± 3 spikes; *t*_23_ = 5.2, *p* < 0.0001), a significant decrease in peak action potential firing frequency (baseline, 4.0 ± 0.5 Hz, 5-HT, 1.9 ± 0.5 Hz; *t*_22_ = 6.0, *p* < 0.0001), and a significant decrease in the duration of action potential firing (baseline, 6.3 ± 0.9 s; 5-HT, 2.1 ± 0.6 s; *t*_24_ = 6.3, *p* < 0.0001). These results show that 5-HT can impair the ability of endogenous ACh to stimulate layer 6 pyramidal neurons effectively.

To probe further the interaction between cholinergic and serotonergic stimulation of layer 6, we investigated how the effects of strong exogenous cholinergic stimulation would be affected by strong exogenous serotonergic stimulation. Bath application of ACh (1 mM) strongly depolarized layer 6 pyramidal neurons, evoking action potential firing from rest. This cholinergic modulation was significantly suppressed by bath application of 5-HT (10 μM). Significant differences were observed in the number of action potentials evoked (baseline, 142 ± 34 spikes, 5-HT, 28 ± 9 spikes; *t*_11_ = 4.0, *p* = 0.0021), in peak firing frequency (baseline, 6.9 ± 1.1 Hz; 5-HT, 2.6 ± 0.9 Hz; *t*_11_ = 7.4, *p* < 0.0001), and in response duration (baseline, 62.4 ± 5.2 s; 5-HT, 35.9 ± 4.0 s; *t*_12_ = 5.27, *p* = 0.0003; Figures 3D,E, *n* = 13). This provides evidence that even the excitation elicited in layer 6 pyramidal neurons by strong cholinergic stimulation can be reduced by serotonergic stimulation, which may be relevant for understanding interactions that may occur when both neuromodulators are increased in prefrontal cortex during highly stressful situations (Mark et al., 1996; Bland et al., 2003).

## Discussion

Here, we probed the opposition between cholinergic and serotonergic modulation of layer 6 pyramidal neurons of prefrontal cortex. First, we examined the effects of ACh and 5-HT on individual pyramidal neurons, then we examined how the excitability of layer 6 neurons is altered by optogenetic release of ACh and 5-HT, and finally we tested the effects of ACh and 5-HT in combination. We used a number of experimental paradigms in this work, including a new paradigm to assess the ability of neuromodulators to enhance or suppress action potential firing in response to a train of brief depolarizing stimuli. Overall, this work shows that individual layer 6 pyramidal neurons in prefrontal cortex are sensitive to direct modulation by both ACh and to 5-HT. This cholinergic excitation and serotonergic inhibition is recapitulated by release of the endogenous neuromodulators. Endogenous excitation of layer 6 pyramidal neurons by ACh was sensitive to both nicotinic and muscarinic receptor blockade, while suppression of endogenously released 5-HT was largely eliminated by blockade of 5-HT_1A_ receptors. We have also shown that the ability of ACh to drive layer 6 pyramidal neurons in prefrontal cortex is constrained by 5-HT. Since cholinergic modulation of deep prefrontal cortex is essential for optimal attentional performance (Bailey et al., 2010; Guillem et al., 2011), these results point to a cellular mechanism to explain how increasing 5-HT release disrupts task attention.

We probed layer 6 pyramidal neurons with lower concentrations of exogenous agonists than typically used in previous work (Kassam et al., 2008; Bailey et al., 2010; Tian et al., 2011, 2016), and used optogenetic stimulation to investigate the consequences of endogenous release of ACh or 5-HT. In working with brain slices from transgenic mice expressing channelrhodopsin in either cholinergic or serotonergic neurons, however, it becomes evident that the expression level of channelrhodopsin in these modulatory afferents determines the maximal effects that can be detected. In short, failure to observe modulation does not mean that it does not happen *in vivo*, just that there may be insufficient potential for the light in the terminal field to stimulate release. This occurs with the Pet1cre/Ai32^+/−^ mouse and may account for the relatively modest effects of optogenetic stimulation in the brain slices of Pet1cre/Ai32^+/+^ mice. We see more powerful effects of optogenetic stimulation in both the ChAT-ChR2 and ChATcre/Ai32^+/−^ mouse lines, which we used to observe the effects of endogenous release of ACh. Our work is a first demonstration of the effects of endogenous serotonergic modulation of prefrontal layer 6, and it extends earlier cholinergic work (Hedrick and Waters, 2015) with experiments assessing the impact of endogenous cholinergic modulation on action potential frequency in response to trains of stimuli delivered to neurons otherwise at rest.

*In vivo*, the attention-enhancing effects of prefrontal ACh release have been extensively studied (Arnold et al., 2002; Parikh et al., 2007; Gritton et al., 2016), but the adverse behavioral effects of 5-HT on attention tasks are less well understood (Ramaekers et al., 1995; Riedel et al., 1999, 2005; Schmitt et al., 2002; Wingen et al., 2008; Golub et al., 2017). Recent work raises the concept that 5-HT may subvert top-down cortical signaling in favor of sensory processing (Lottem et al., 2016). This hypothesis is consistent with the inhibitory effects of 5-HT on layer 6 neurons known for their top-down feedback to the thalamus (Alitto and Usrey, 2003; Zikopoulos and Barbas, 2006; Thomson, 2010) and role in cortical gain control (Olsen et al., 2012; Tian et al., 2016). While both the cholinergic and serotonergic systems are active during waking (Buzsaki et al., 1988; Jacobs and Azmitia, 1992) and the cholinergic system is certainly active during attention (Arnold et al., 2002; Parikh et al., 2007; Gritton et al., 2016), the cholinergic modulation of prefrontal cortex may be opposed by 5-HT to differing extents depending on the environmental circumstances (Ranade and Mainen, 2009), such as the presence of stressors (Fujino et al., 2002; Bland et al., 2003) or potentially the emotional content of an attention task (Osinsky et al., 2008; Daly et al., 2010; Beacher et al., 2011; Elliott et al., 2011; Frodl et al., 2015).

What would be the benefit of 5-HT attenuating the cholinergic enhancement of task attention? While our society rewards strong task attention, interference by 5-HT appears consistent with the growing understanding of serotonergic modulation of cognitive and behavioral flexibility (Nonkes et al., 2012; Matias et al., 2017). Clinically, this phenomenon appears relevant to attention abnormalities seen in neurological and psychiatric disorders that are accompanied by serotonergic disruption. For example, some types of focused task attention can be difficult to disrupt in people with autism, a condition associated with low 5-HT levels in brain (Dougherty et al., 2013; Adamsen et al., 2014; Muller et al., 2016). Conversely, increases in 5-HT may contribute to adverse consequences of selective 5-HT reuptake inhibitors on attention (Ramaekers et al., 1995; Riedel et al., 2005; Graf et al., 2013; Golub et al., 2017). Taken together, our electrophysiological and optogenetic results suggest a potential cellular mechanism underlying the opposing influences of ACh and 5-HT on attention: these modulators exert opposite neurophysiological effects on the excitability of layer 6 pyramidal neurons in prefrontal cortex.

## Author Contributions

DWS, MKT, DS, SV, KI and EKL designed the experiments; contributed to the revision of the article. DWS, MKT, DS, KI and SV performed the experiments and analyzed the data. DWS, MKT and EKL wrote the article.

## Conflict of Interest Statement

The authors declare that the research was conducted in the absence of any commercial or financial relationships that could be construed as a potential conflict of interest.
